# Comparison of Postoperative MELD‐Based Scores in Predicting Mortality After Liver Transplantation: A Retrospective Comparative Study

**DOI:** 10.1155/ijh/3307874

**Published:** 2026-05-18

**Authors:** Akira Katayama, Ezeldeen Abuelkasem, David W. Wang

**Affiliations:** ^1^ Department of Anesthesiology and Perioperative Medicine, University of Pittsburgh, Pittsburgh, Pennsylvania, USA, pitt.edu; ^2^ Department of Anesthesiology and Resuscitology, Okayama University Graduate School of Medicine, Dentistry and Pharmaceutical Sciences, Okayama, Japan, okayama-u.ac.jp; ^3^ Department of Critical Care Medicine, University of Pittsburgh, Pittsburgh, Pennsylvania, USA, pitt.edu

**Keywords:** liver transplantation, Model for End-Stage Liver Disease, mortality, risk stratification

## Abstract

**Background:**

Postoperative prognostic assessment is critical for patients undergoing liver transplantation (LT). The Model for End‐Stage Liver Disease (MELD) scores calculated in the early postoperative period have been shown to be associated with postoperative outcomes. While MELD‐Na and MELD 3.0 were developed to improve mortality prediction in pretransplant settings by incorporating serum sodium, albumin, and sex, their comparative utility in the postoperative period remains unclear.

**Methods:**

We conducted a retrospective single‐center cohort study including adult LT recipients between January 2012 and June 2023. MELD, MELD‐Na, and MELD 3.0 scores were calculated on Postoperative Day (POD) 7. The primary outcome was 30‐day all‐cause mortality. Predictive performance was assessed using the area under the receiver operating characteristic (ROC) curve (AUC). Subgroup analyses were performed according to serum sodium, albumin levels, and sex.

**Results:**

A total of 1038 patients were included. All three MELD‐based scores calculated on POD 7 were associated with 30‐day mortality (MELD original, hazard ratio [HR] 1.20, 95% CI 1.13–1.27, *p* < 0.001; MELD‐Na, HR 1.20, 95% CI 1.12–1.28, *p* < 0.001; and MELD 3.0, HR 1.21, 95% CI 1.14–1.29, *p* < 0.001, respectively). ROC analysis demonstrated comparable predictive accuracy across MELD, MELD‐Na, and MELD 3.0 for 30‐day mortality (AUCs: MELD original, 0.82; MELD‐Na, 0.79; MELD 3.0, 0.82; overall *p* = 0.24). Exploratory subgroup analyses did not identify any score with consistently superior performance across different strata of sodium, albumin, or sex.

**Conclusion:**

MELD, MELD‐Na, and MELD 3.0 scores calculated on POD 7 showed comparable performance in predicting 30‐day mortality after LT. These findings support the use of POD 7 MELD‐based scores for postoperative risk stratification, with no meaningful difference in predictive performance among the three scoring systems.

## 1. Introduction

The Model for End‐Stage Liver Disease (MELD) score was first developed to predict short‐term mortality in patients undergoing transjugular intrahepatic portosystemic shunt (TIPS) procedures in 2000 [1]. MELD score has since been widely used after it was adopted by the United Network for Organ Sharing (UNOS) in 2002 to prioritize patients on the liver transplant waiting list in the United States. Subsequently, after hyponatremia was found to be a strong predictor of mortality in patients with liver disease, the MELD‐Na score was newly adopted in 2016 to improve the predictive accuracy of the MELD original score by incorporating serum sodium [2]. Furthermore, MELD 3.0 was developed in 2021 to address known limitations of the MELD and MELD‐Na scores, particularly the underestimation of disease severity in women and patients with low serum albumin [3].

Despite its original use as a pretransplant prognostic tool, the MELD score has gained attention as a dynamic postoperative marker that may reflect postoperative outcomes after liver transplantation (LT). MELD score calculated postoperatively has been found to be a predictor for postoperative complications, early allograft failure, and mortality after LT [4–7]. While MELD, MELD‐Na, and MELD 3.0 have been extensively compared as prognostic tools for patients with cirrhosis [8, 9], their relative performances have not been evaluated in the postoperative setting. Thus, it remains unclear which MELD‐based scoring system provides the most accurate prediction of outcomes when calculated after transplantation.

This study is aimed at comparing the postoperative MELD original, MELD‐Na, and MELD 3.0 as predictive values for post‐LT 30‐day mortality.

## 2. Materials and Methods

### 2.1. Study Design and Patients

This was a retrospective cohort study including all adult recipients of orthotopic liver transplants performed at the University of Pittsburgh Medical Center, United States, from January 2012 to June 2023. Exclusion criteria were as follows: (1) intraoperative death, (2) simultaneous transplantation, (3) dependency on hemodialysis, (4) patients with missing data required for MELD score calculation (i.e., serum creatinine, bilirubin, INR, sodium, or albumin values preoperatively or within 7 postoperative days), and (5) insufficient anesthetic or preoperative laboratory records. The study was approved by the University of Pittsburgh institutional review board (STUDY20050148) and conducted in accordance with the Declaration of Helsinki. The requirement for written informed consent was waived by the institutional review board owing to the retrospective design of the study. This study adheres to the Strengthening the Reporting of Observational Studies in Epidemiology (STROBE) statement of reporting observational studies [10].

The following data were collected for all subjects: demographics including age, sex, and body mass index (BMI); presence of hypertension or diabetes mellitus (DM); etiology of liver failure, preoperative laboratory findings within 1 week prior to LT; and MELD score. Data for the duration of the procedure, volume of infusion, and blood loss were recorded as intraoperative factors. We also investigated allograft information including donor age, cold ischemic time (CIT), and warm ischemic time (WIT).

### 2.2. Calculation of MELD Score

The MELD original, MELD‐Na, and MELD 3.0 scores were calculated according to the following formula:

*M* *E* *L* *D* *o* *r* *i* *g* *i* *n* *a* *l* [1] = 9.57 × log_*e*_(*c* *r* *e* *a* *t* *i* *n* *i* *n* *e*) + 3.78 × log_*e*_(*b* *i* *l* *i* *r* *u* *b* *i* *n*) + 11.20 × log_*e*_(*I* *N* *R*) + 6.43. The values of serum creatinine, bilirubin, and INR less than one were given a value of 1. The upper limit of serum creatinine was set to 4.0 mg/dL.

*M* *E* *L* *D* − *N* *a* [3] = (*M* *E* *L* *D* *o* *r* *i* *g* *i* *n* *a* *l*) + [1.32 × (137 − *N* *a*)] − [0.033 × (*M* *E* *L* *D* *o* *r* *i* *g* *i* *n* *a* *l*) × (137 − *N* *a*)]. The lower and upper limits of serum sodium are 125 and 137 mmol/L, respectively.

*M* *E* *L* *D* 3.0 [3] = 1.33 (*if* *f* *e* *m* *a* *l* *e*) + [4.56 × log_*e*_(*b* *i* *l* *i* *r* *u* *b* *i* *n*)] + [0.82 × (137 − *N* *a*)] − [0.24 × (137 − *N* *a*) × log_*e*_(*b* *i* *l* *i* *r* *u* *b* *i* *n*)] + [9.09 × log_*e*_(*I* *N* *R*)] + [11.14 × log_*e*_(*c* *r* *e* *a* *t* *i* *n* *i* *n* *e*)] + [1.85 × (3.5 − *a* *l* *b* *u* *m* *i* *n*)] − [1.83 × (3.5 − *a* *l* *b* *u* *m* *i* *n*) × log_*e*_(*c* *r* *e* *a* *t* *i* *n* *i* *n* *e*)] + 6. The values of serum creatinine, bilirubin, and INR less than one were given a value of 1. The lower and upper limits of serum sodium are 125 and 137 mmol/L, respectively. The upper limit of serum creatinine was set to 3.0 mg/dL. The lower and upper limits of serum albumin are 1.5 and 3.5 g/dL, respectively.

### 2.3. Outcomes

The primary outcome was 30‐day mortality after LT. We evaluated the individual performances of MELD original, MELD‐Na, and MELD 3.0 to predict 30‐day mortality at POD 7. Subgroup analyses were performed to explore outcome differences based on serum sodium, albumin levels, and sex.

### 2.4. Statistical Analysis

The results were analyzed using StataSE Version 17.0 (College Station, Texas, United States) and EZR (Saitama Medical Center, Jichi Medical University, Saitama, Japan), a graphical user interface for R Version 4.4.1 (R Foundation for Statistical Computing, Vienna, Austria). Continuous variables are presented as median with interquartile ranges (IQRs) and categorical variables as numbers (%). Baseline characteristics were summarized descriptively without between‐group testing. Comparisons of clinical variables such as postoperative MELD scores between survivors and nonsurvivors were performed using the Mann–Whitney *U* test, as appropriate.

To determine the optimal timing for postoperative MELD score assessment, we conducted receiver operating characteristic (ROC) curve analysis for 30‐day mortality and compared the area under the ROC curves (AUCs) of MELD original score calculated on POD 1, 3, 5, and 7 using the DeLong test. Spearman’s rank correlation coefficients were calculated to assess the linear relationships among MELD original, MELD‐Na, and MELD 3.0 scores. In order to identify perioperative risk factors for post‐LT mortality, we performed univariable Cox proportional hazards regression analyses using the following known risk factors for post‐LT mortality: age, sex, BMI, preoperative MELD score, DM, and intraoperative blood loss [11–16]. The ROC curve analysis was conducted to evaluate and compare the predictive performance of MELD original, MELD‐Na, and MELD 3.0 scores for post‐LT 30‐day mortality. Discriminative performance for 30‐day mortality was assessed using the AUC. The AUC was calculated for each score, and pairwise comparisons between AUCs were performed using the DeLong test. For three prespecified pairwise comparisons, Bonferroni‐adjusted significance was defined as *p* < 0.017. Since MELD‐Na and MELD 3.0 were developed to address disparities related to sex, hyponatremia, and hypoalbuminemia, subgroup analyses were performed based on these variables. For sodium, patients were categorized into a normal sodium group (≥ 135 mmol/L) and a hyponatremia group (< 135 mmol/L). For albumin, patients were classified into a normal albumin group (≥ 3.5 g/dL), a mild hypoalbuminemia group (3.0–3.49 g/dL), a moderate hypoalbuminemia group (2.5–2.99 g/dL), and a severe hypoalbuminemia group (< 2.5 g/dL).

## 3. Results

Of the 1148 adult patients who underwent LT between January 2012 and June 2023, 8 were excluded due to intraoperative death, 34 due to simultaneous transplantation, 32 due to dependence on hemodialysis, 29 due to the lack of data to calculate MELD score, and 7 due to insufficient anesthetic or preoperative laboratory records, resulting in a final study cohort of 1038 patients. A total of 18 patients died within 30 days after LT, and the 30‐day mortality rate based on Kaplan–Meier estimates was 1.7%.

Comparison of demographic characteristics, preoperative laboratory findings, and intraoperative variables between 30‐day nonsurvivors and survivors is summarized in Table 1. Overall, patient demographics and preoperative laboratory values were broadly similar between survivors and nonsurvivors. Nonsurvivors tended to have slightly lower BMI and platelet counts, and they required larger transfusion volumes during surgery. Other perioperative variables showed comparable distributions.

**Table 1:** Baseline characteristics and perioperative variables of the study cohort stratified by 30‐day survival status.

	All patients *n* = 1038	30‐day survivors *n* = 1020	30‐day nonsurvivors *n* = 18
Age (years)	58 [50, 64.8]	58 [50, 65]	58 [43, 64]
Sex, male, *n* (%)	699 (66.4%)	690 (67.7%)	9 (50.0%)
BMI (kg/m^2^)	28.4 [25.0, 33.1]	28.4 [25.0, 33.2]	26.9 [22.4, 28.9]
Etiology of liver failure			
Alcoholic, *n* (%)	252 (24.3%)	250 (24.5%)	2 (11.1%)
NASH, *n* (%)	229 (22.1%)	226 (22.2%)	3 (16.7%)
HCC, *n* (%)	302 (29.2%)	300 (29.5%)	2 (11.1%)
Hypertension, *n* (%)	524 (50.5%)	515 (50.5%)	9 (50.0%)
DM II, *n* (%)	352 (33.9%)	344 (33.7%)	8 (44.4%)
MELD original	17 [13, 24]	17 [13, 24]	15.5 [12, 28]
MELD‐Na	19 [14, 27]	19 [14, 27]	16 [15, 28]
MELD 3.0	20 [14, 27]	20 [14, 27]	18 [14, 30]
Donor age, years	40 [30, 49]	40 [30, 49]	37.5 [32, 48]
Donor type (living donor), *n* (%)	547 (52.7%)	535 (52.5%)	12 (66.7%)
AST (U/L)	47 [33, 72]	47 [33, 72]	61.5 [36, 76]
Albumin (g/dL)	3.2 [2.9, 3.7]	3.2 [2.9, 3.7]	3.3 [2.7, 3.7]
Bilirubin (mg/dL)	2.7 [1.4, 6.2]	2.7 [1.4, 6.2]	2.7 [1.0, 8.0]
Platelet count (×10^9^/L)	8.3 [5.7, 12.7]	8.3 [5.7, 12.7]	6.6 [4.4, 13.9]
PT‐INR	1.6 [1.4, 2.1]	1.6 [1.4, 2.1]	1.7 [1.3, 2.3]
Creatinine (mg/dL)	1.0 [0.8, 1.4]	1.0 [0.8, 1.4]	1.0 [0.8, 1.2]
Sodium (mmol/L)	136 [132, 138]	135 [132, 138]	137 [134, 138]
HbA1c (%)	5.1 [4.5, 6.1]	5.1 [4.5, 6.0]	6.7 [4.3, 7.9]
Duration of surgery (min)	481 [408, 572.8]	480 [407, 569]	590 [521, 745]
CIT (min)	160.5 [110.0, 412.8]	161 [110, 415]	140 [95, 322]
WIT (min)	30 [25, 36]	30 [25, 36]	32 [25, 43]
Blood loss (mL)	800 [500, 1500]	800 [500, 1500]	1150 [600, 4000]
RBC (mL)	500 [0, 1200]	500 [0, 1200]	1350 [300, 2000]
Plasma (mL)	0 [0, 800]	0 [0, 800]	591 [0, 1600]
PC (mL)	0 [0, 291]	0 [0, 284]	245 [0, 520]
Colloid (mL)	1250 [750, 2000]	1250 [750, 2000]	2000 [1000, 3000]

Abbreviations: ALT, alanine transaminase; AST, aspartate aminotransferase; BMI, body mass index; CIT, cold ischemic time; DM, diabetes mellitus; HCC, hepatic cell cancer; MELD, model for end‐stage liver disease; NASH, nonalcoholic steatohepatitis; PC, platelet concentrate; RBC, red blood cell; WIT, warm ischemic time.

**Figure 1:**
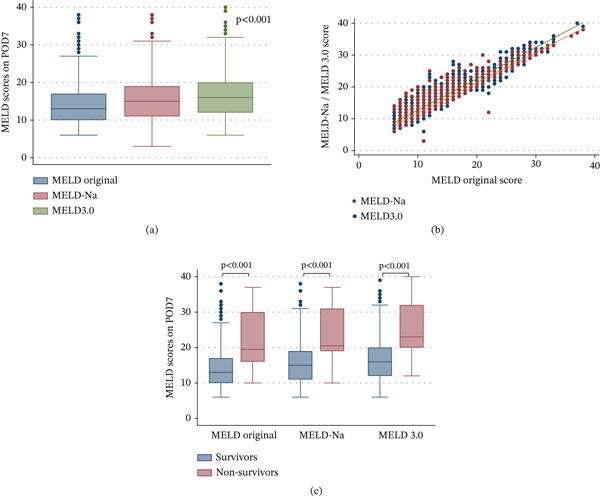
(A) Distribution of MELD scores on POD 7. (B) Correlation between MELD original, MELD‐Na, and MELD 3.0 scores on POD 7. (C) Postoperative Day 7 MELD‐based scores in survivors and nonsurvivors after liver transplantation. Abbreviations: LT, liver transplantation; MELD, model for end‐stage liver disease; POD, postoperative day.

Comparison of MELD scores on POD 1, 3, 5, and 7 between survivors and nonsurvivors and their predictive performance for 30‐day mortality is summarized in Table 2. MELD scores on POD 1, 3, 5, and 7 were all significantly higher in nonsurvivors than in survivors (*p* = 0.02, *p* = 0.002, *p* < 0.001, and *p* < 0.001, respectively). Among MELD scores calculated on POD 1, 3, 5, and 7, the MELD score on POD 7 demonstrated the highest AUC for predicting 30‐day mortality (*A* *U* *C* = 0.82), and the differences among the time points were statistically significant (*p* = 0.02). Based on these findings, we selected POD 7 MELD scores for further comparative analyses with MELD‐Na and MELD 3.0. The median MELD original, MELD‐Na, and MELD 3.0 calculated on POD 7 were 13, 15, and 16, respectively, and there was a significant difference among the scores (*p* < 0.001) (Figure 1A). Figure 1B shows the correlation between MELD original, MELD‐Na, and MELD 3.0 scores on POD 7. All three scores were highly correlated with each other, with Spearman correlation coefficients of 0.92 between MELD original and MELD‐Na, 0.94 between MELD original and MELD 3.0, and 0.97 between MELD‐Na and MELD 3.0. MELD original, MELD‐Na, and MELD 3.0 scores were all higher in the nonsurvivors compared to survivors (MELD original: 19.5 vs. 13, *p* < 0.001; MELD‐Na: 20.5 vs. 15, *p* < 0.001; and MELD 3.0: 23 vs. 16, *p* < 0.001, respectively) (Figure 1C).

**Table 2:** MELD scores on POD 1, 3, 5, and 7 in survivors and nonsurvivors and their predictive performance for 30‐day mortality.

	Survivor	Nonsurvivor	*p* value	AUC	95% CI	*p* value for AUCs
MELD POD 1	22 [18, 26]	27 [21, 30]	0.02	0.66	0.54‐0.79	0.02
MELD POD 3	16 [12, 21]	20 [18, 23]	0.002	0.71	0.63–0.80	
MELD POD 5	14 [11, 18]	21 [16, 27]	< 0.001	0.79	0.69–0.88	
MELD POD 7	13 [10, 17]	19.5 [16, 30]	< 0.001	0.82	0.72–0.91	

*Note:* Values are presented as median [IQR]. The *p* values compare survivors and nonsurvivors using the Mann–Whitney *U* test. AUCs were derived from ROC analysis to evaluate the discriminative performance of each MELD‐based score for predicting 30‐day mortality.

Abbreviations: AUC, area under the receiver operating characteristic curve; MELD, model for end‐stage liver disease; POD, postoperative day.

**Figure 2:**
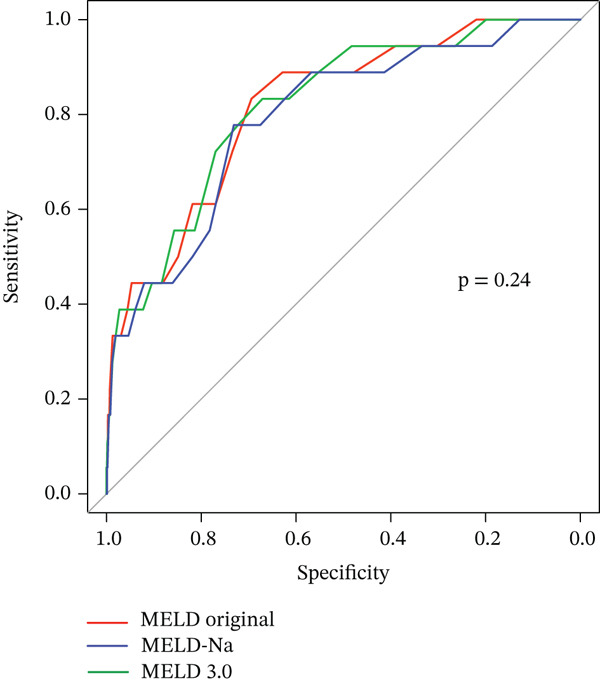
ROC curves comparing MELD, MELD‐Na, and MELD 3.0 scores on POD 7 for predicting mortality at 30 days. Abbreviations: MELD, model for end‐stage liver disease; POD, postoperative day; ROC, receiver operating characteristic.

Univariable Cox analyses for 30‐day mortality are presented in Table 3. Among baseline and perioperative variables, greater intraoperative blood loss was associated with an increased risk of 30‐day mortality. In addition, all MELD‐based scores measured on POD 7 showed significant associations with 30‐day mortality, with hazard ratios ranging from 1.20 to 1.21 per point increase. Other demographic and preoperative variables did not demonstrate clear associations with early mortality. ROC analysis demonstrated good discriminative performance for all three MELD‐based scores (Figure 2). AUCs were similar across MELD original (AUC, 0.82, 95% CI 0.72–0.91), MELD‐Na (AUC, 0.79, 95% CI 0.68–0.90), and MELD 3.0 (AUC, 0.82, 95% CI 0.72–0.92), with no statistically significant differences in pairwise comparisons (Table 4).

**Table 3:** Univariable Cox regression analysis for 30‐day mortality.

Variables	Univariable analysis		
	HR	95% CI	*p* value
Age, per 10‐year increase	0.94	0.64–1.38	0.74
Sex, male	0.48	0.19–1.21	0.12
BMI (kg/m^2^)	0.94	0.86–1.02	0.13
DM II	1.57	0.62–3.97	0.35
Preoperative MELD original score	0.98	0.93–1.03	0.42
Intraoperative blood loss (L)	1.19	1.11–1.28	< 0.001
MELD POD 7	1.20	1.13–1.27	< 0.001
MELD‐Na POD 7	1.20	1.12–1.28	< 0.001
MELD 3.0 POD 7	1.21	1.14–1.29	< 0.001

*Note:* Hazard ratios (HRs) and 95% confidence intervals (CIs) were calculated using univariable Cox proportional hazards models for each variable. MELD‐based scores were assessed on Postoperative Day 7.

Abbreviations: BMI, body mass index; CI, confidence interval; DM, diabetes mellitus; HR, hazard ratio; MELD, model for end‐stage liver disease; POD, postoperative day.

**Table 4:** Pairwise differences in AUC among MELD‐based scores.

Pairwise comparison	*Δ*AUC	95% CI (bootstrap)	*p* value (DeLong)
MELD original–MELD‐Na	−0.02	−0.05 to 0.006	0.12
MELD original–MELD 3.0	0.002	−0.03 to 0.03	0.92
MELD‐Na–MELD 3.0	−0.03	−0.06 to 0.008	0.14

*Note:* Pairwise comparison of the areas under the receiver operating characteristic (ROC) curves (AUCs) among MELD‐based scores was performed using the DeLong test. Bonferroni correction for three pairwise comparisons yields a significance threshold of *p* < 0.017.

Abbreviations: AUC, area under the receiver operating characteristic curve; CI, confidence interval; MELD, model for end‐stage liver disease.

**Figure 3:**
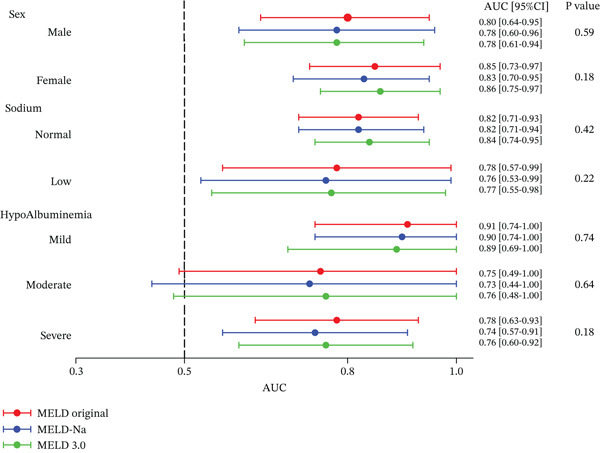
Subgroup analysis comparing the predictive performance of MELD, MELD‐Na, and MELD 3.0 scores for 30‐day mortality according to sex, serum sodium, and albumin levels. Abbreviations: AUC, area under the curve; MELD, model for end‐stage liver disease.

Subgroup analyses by sex, sodium category, and albumin status yielded results that were consistent with the overall cohort (Figure 3). The normal albumin group was excluded from the subgroup analysis because no deaths occurred in this group. In every subgroup, the AUCs for MELD original, MELD‐Na, and MELD 3.0 were similar, and no significant differences were detected in pairwise AUC comparisons, indicating comparable discriminative performance across patient strata.

## 4. Discussion

Although MELD original, MELD‐Na, and MELD 3.0 were originally developed to predict mortality in patients awaiting LT, their prognostic performance in the postoperative setting has not been thoroughly evaluated. In this retrospective cohort study, we evaluated the prognostic performance of these three MELD‐based scoring systems calculated on POD 7 in patients undergoing LT. All three scores demonstrated both similar and significant predictive ability for 30‐day mortality. Subgroup analyses stratified by sex, serum sodium, and albumin levels did not reveal meaningful differences in the relative performance of the three MELD‐based scores.

In our study, MELD scores calculated on POD 7 showed a significant association with 30‐day mortality following LT, and the MELD score demonstrated good discriminative ability with an AUC over 0.8. These findings indicate that postoperative MELD scores reflect early postoperative physiological status and may provide meaningful prognostic information beyond their traditional role as pretransplant risk indicators. It is important to note that MELD‐based scores calculated in the postoperative period should not be interpreted as direct measures of intrinsic graft function. Rather, these scores reflect a composite of recipient physiological status, including liver function, coagulation, and renal dysfunction, and may therefore serve as integrated markers of early postoperative recovery. In fact, several studies have identified that the MELD score calculated postoperatively was a predictive factor for post‐LT complications, early allograft dysfunction, and mortality [4–7]. Furthermore, Wong et al. demonstrated that the MELD score on POD 7 most accurately predicted 1‐year mortality after LT, which is consistent with the findings of our study [17]. Our findings highlight the importance of reassessing patient risk in the early postoperative period using the MELD score, as clinical status can change rapidly from day to day despite the presence of established preoperative and intraoperative prognostic factors. These findings may have practical clinical implications, as postoperative MELD‐based scores could serve as simple and readily available tools for early risk stratification and may assist clinicians in identifying high‐risk patients who require closer monitoring or intensified postoperative management.

A key finding of this study was that the three MELD‐based scoring systems—MELD original, MELD‐Na, and MELD 3.0—were highly correlated with one another and demonstrated comparable predictive performance of postoperative mortality. Despite their structural differences and the inclusion of additional variables in MELD‐Na and MELD 3.0, none of the modified scores showed a statistically significant improvement over the original MELD score in predicting 30‐day mortality. In patients with cirrhosis, several studies have shown that MELD 3.0 was a statistically superior prognostic model compared to MELD original and MELD‐Na [8, 18, 19]. The superior predictive performance of MELD 3.0 is likely attributable to its incorporation of additional clinically relevant factors beyond those included in MELD‐Na—namely, serum albumin, which reflects nutritional and inflammatory status, and adjustment for female sex, which mitigates the gender‐based bias inherent in creatinine‐based assessments [2, 3, 18]. Although serum sodium is also included in MELD‐Na, its presence in MELD 3.0, in combination with these other variables, may contribute to a more comprehensive assessment of disease severity. These modifications enhance the score’s ability to capture patient‐specific risk more accurately, particularly in those with severe liver dysfunction, hyponatremia, or hypoalbuminemia. In the setting of post‐LT, however, no previous studies have compared the predictive value of postoperative MELD 3.0 with MELD original or MELD‐Na, whereas prior research comparing postoperative MELD original and MELD‐Na has shown that their predictive performances were similar [20, 21]. The lack of a statistically significant difference in predictive performance among MELD original, MELD‐Na, and MELD 3.0 in our study may be attributed to the fact that serum sodium and albumin levels measured on POD 7 were strongly influenced by perioperative management, including fluid therapy and nutritional support. These variables may not necessarily reflect the underlying physiological derangements, resulting in the inability to clearly demonstrate the advantage of MELD 3.0 over the other models.

Given that MELD‐Na and MELD 3.0 were developed to address prognostic disparities related to hyponatremia, hypoalbuminemia, and female sex, we performed subgroup analyses based on serum sodium, albumin levels, and sex. Across all subgroups, however, the discriminative performance of MELD original, MELD‐Na, and MELD 3.0 remained broadly similar, and no statistically significant differences were observed. These findings suggest that score modifications intended to improve prediction in specific pretransplant populations may not translate into enhanced prognostic ability in the early postoperative setting. As this study represents the first comparison of postoperative MELD‐based scores for short‐term mortality after LT, further investigations are warranted to clarify whether postoperative risk stratification can be refined for particular patient subgroups.

Several previous studies have demonstrated associations between postoperative mortality and other prognostic models such as Acute Physiology and Chronic Health Evaluation (APACHE) score and Sequential Organ Failure Assessment (SOFA) score [17, 21–23]. Angus et al. found that the postoperative APACHE II score was strongly associated with mortality [22], and Lee et al. demonstrated good discrimination of the APACHE IV score for early post‐LT outcomes [21]. Similarly, the SOFA score calculated on POD 7 showed good discriminative ability for predicting mortality after LT [17, 23]. These models incorporate numerous physiologic variables and are advantageous in their ability to reflect overall postoperative clinical status. However, both APACHE and SOFA scores require many parameters and are relatively complex to calculate and neither is specific to liver disease. Prior studies have shown that the MELD score has predictive performance comparable to that of these more complex models [24, 25]. In this context, our findings further support the potential role of postoperative MELD assessment as a practical and informative tool for early risk prediction after LT.

This study has several limitations that should be acknowledged. First, it was a single‐center retrospective analysis, which may limit the generalizability of the findings to other institutions or populations with different perioperative management protocols. Second, although MELD scores were calculated using standardized laboratory values on Postoperative Day 7, these values may have been influenced by perioperative interventions such as fluid resuscitation, diuretics, and albumin administration, potentially altering the prognostic value of sodium and albumin. Third, the number of early mortality events was relatively small, which reduces the precision of risk estimates and increases statistical uncertainty, particularly for analyses focused on short‐term mortality. In addition, the use of 30‐day mortality as the primary outcome may have further limited the number of events and may not fully capture longer term outcomes. Fourth, while we compared the predictive performance of three MELD‐based models, the study was not powered to detect small differences in AUC between models. Therefore, the absence of statistically significant differences should be interpreted with caution, as subtle performance distinctions may not have been detectable with the available sample size. Finally, we did not perform multivariable adjustment to account for potential confounding factors. Although the main focus of this study was to evaluate the discriminative performance of MELD‐based scores themselves, residual confounding cannot be excluded. Further large‐scale studies will be essential to validate these results and to define the optimal application of postoperative MELD‐based scores in clinical practice.

In conclusion, MELD, MELD‐Na, and MELD 3.0 scores calculated on Postoperative Day 7 demonstrated comparable performance in predicting 30‐day mortality following LT. Although MELD‐Na and MELD 3.0 were designed to improve risk stratification through the inclusion of sodium, albumin, and female sex, these advantages were not clearly observed in the postoperative setting. These findings suggest that postoperative MELD assessment may provide useful prognostic information, but further validation in larger and more diverse cohorts is warranted.

## Funding

No funding was received for this manuscript.

## Conflicts of Interest

The authors declare no conflicts of interest.

## Data Availability

The data that support the findings of this study are available on request from the corresponding author. The data are not publicly available due to privacy or ethical restrictions.

## References

[1] Malinchoc M. , Kamath P. S. , Gordon F. D. , Peine C. J. , Rank J. , and ter Borg P. C. , A Model to Predict Poor Survival in Patients Undergoing Transjugular Intrahepatic Portosystemic Shunts, Hepatology. (2000) 31, no. 4, 864–871, 10.1053/he.2000.5852, 2-s2.0-0034106289, 10733541. 10733541

[2] Biggins S. W. , Kim W. R. , Terrault N. A. , Saab S. , Balan V. , Schiano T. , Benson J. , Therneau T. , Kremers W. , Wiesner R. , Kamath P. , and Klintmalm G. , Evidence-Based Incorporation of Serum Sodium Concentration Into MELD, Gastroenterology. (2006) 130, no. 6, 1652–1660, 10.1053/j.gastro.2006.02.010, 2-s2.0-33646372427, 16697729. 16697729

[3] Kim W. R. , Mannalithara A. , Heimbach J. K. , Kamath P. S. , Asrani S. K. , Biggins S. W. , Wood N. L. , Gentry S. E. , and Kwong A. J. , MELD 3.0: The Model for End-Stage Liver Disease Updated for the Modern Era, Gastroenterology. (2021) 161, no. 6, 1887–1895.e4, 10.1053/j.gastro.2021.08.050, 34481845. 34481845 PMC8608337

[4] Pommergaard H. C. , Daugaard T. R. , Rostved A. A. , Schultz N. A. , Hillingsø J. , Krohn P. S. , and Rasmussen A. , Model for End-Stage Liver Disease Score Predicts Complications After Liver Transplantation, Langenbeck′s Archives of Surgery. (2021) 406, no. 1, 55–65, 10.1007/s00423-020-02018-3. 33140185

[5] Wagener G. , Raffel B. , Young A. T. , Minhaz M. , and Emond J. , Predicting Early Allograft Failure and Mortality After Liver Transplantation: The Role of the Postoperative Model for End-Stage Liver Disease Score, Liver Transplantation. (2013) 19, no. 5, 534–542, 10.1002/lt.23634, 2-s2.0-84876944041, 23576469. 23576469

[6] Dashti H. , Ebrahimi A. , Khorasani N. R. , Moazzami B. , Khojasteh F. , Shabanan S. H. , and Jafarian A. , The Utility of Early Post-Liver Transplantation Model for End-Stage Liver Disease Score in Prediction of Long-Term Mortality, Annals of Gastroenterology. (2019) 32, no. 6, 633–641, 10.20524/aog.2019.0420, 31700242. 31700242 PMC6826064

[7] Rostved A. A. , Lundgren J. D. , Hillingsø J. , Peters L. , Mocroft A. , and Rasmussen A. , MELD Score Measured Day 10 After Orthotopic Liver Transplantation Predicts Death and Re-Transplantation Within the First Year, Scandinavian Journal of Gastroenterology. (2016) 51, no. 11, 1360–1366, 10.1080/00365521.2016.1196497, 2-s2.0-84975223397, 27319374. 27319374

[8] Lin H. Y. , Loi P. L. , Ng J. , Shen L. , Teo W. Q. , Chung A. , Raj P. , and Chang J. P. E. , MELD3.0 Is Superior to MELDNa and MELD for Prediction of Mortality in Patients With Cirrhosis: An External Validation in a Multi-Ethnic Population, JGH Open. (2024) 8, no. 6, e13098, 10.1002/jgh3.13098, 38832135. 38832135 PMC11144281

[9] Yoo J. J. , Chang J. I. , Moon J. E. , Sinn D. H. , Kim S. G. , and Kim Y. S. , Validation of MELD 3.0 Scoring System in East Asian Patients With Cirrhosis Awaiting Liver Transplantation, Liver Transpl.(2023) 29, no. 10, 1029–1040, 10.1097/LVT.0000000000000126, 36929833. 36929833

[10] von Elm E. , Altman D. G. , Egger M. , Pocock S. J. , Gøtzsche P. C. , Vandenbroucke J. P. , and Initiative S. T. R. O. B. E. , The Strengthening the Reporting of Observational Studies in Epidemiology (STROBE) Statement: Guidelines for Reporting Observational Studies, International Journal of Surgery. (2014) 12, no. 12, 1495–1499, 10.1016/j.ijsu.2014.07.013, 2-s2.0-84919384602. 25046131

[11] Chen H. P. , Tsai Y. F. , Lin J. R. , Liu F. C. , and Yu H. P. , Recipient Age and Mortality Risk After Liver Transplantation: A Population-Based Cohort Study, PLoS One. (2016) 11, no. 3, e0152324, 10.1371/journal.pone.0152324, 2-s2.0-84962178580, 27019189. 27019189 PMC4809564

[12] Serrano M. T. , Sabroso S. , Esteban L. M. , Berenguer M. , Fondevila C. , Lorente S. , Cortés L. , Sanchez-Antolin G. , Nuño J. , de la Rosa G. , and Salcedo M. , Mortality and Causes of Death After Liver Transplantation: Analysis of Sex Differences in a Large Nationwide Cohort, Transplant International. (2022) 35, no. 35, 10263, 10.3389/ti.2022.10263, 35615539. 35615539 PMC9124758

[13] Beckmann S. , Drent G. , Ruppar T. , Nikolić N. , and De Geest S. , Body Weight Parameters Are Related to Morbidity and Mortality After Liver Transplantation: A Systematic Review and Meta-analysis, Transplantation. (2019) 103, no. 11, 2287–2303, 10.1097/TP.0000000000002811, 2-s2.0-85074117863, 31283679. 31283679

[14] Watt K. D. , Pedersen R. A. , Kremers W. K. , Heimbach J. K. , and Charlton M. R. , Evolution of Causes and Risk Factors for Mortality Post-Liver Transplant: Results of the NIDDK Long-Term Follow-Up Study, American Journal of Transplantation. (2010) 10, no. 6, 1420–1427, 10.1111/j.1600-6143.2010.03126.x, 2-s2.0-77952902738, 20486907. 20486907 PMC2891375

[15] Zhang X. M. , Fan H. , Wu Q. , Zhang X. X. , Lang R. , and He Q. , In-Hospital Mortality of Liver Transplantation and Risk Factors: A Single-Center Experience, Annals of Translational Medicine. (2021) 9, no. 5, 10.21037/atm-20-5618. PMC8033294 33842590

[16] Jóźwik A. , Karpeta E. , Nita M. , Łągiewska B. , and Pacholczyk M. , Impact of Blood Loss and Intraoperative Blood Transfusion During Liver Transplantation on the Incidence of Early Biliary Complications and Mortality, Transplantation Proceedings. (2020) 52, no. 8, 2477–2479, 10.1016/j.transproceed.2020.03.052, 32434743. 32434743

[17] Wong C. S. , Lee W. C. , Jenq C. C. , Tian Y. C. , Chang M. Y. , Lin C. Y. , Fang J. T. , Yang C. W. , Tsai M. H. , Shih H. C. , and Chen Y. C. , Scoring Short-Term Mortality After Liver Transplantation, Liver Transplantation. (2010) 16, no. 2, 138–146, 10.1002/lt.21969, 2-s2.0-75449097855. 20104481

[18] Song J. , Wang X. , Yan Y. , Xiang T. , and Luo X. , MELD 3.0 Score for Predicting Survival in Patients With Cirrhosis After Transjugular Intrahepatic Portosystemic Shunt Creation, Digestive Diseases and Sciences. (2023) 68, no. 7, 3185–3192, 10.1007/s10620-023-07834-3, 36715817. 36715817

[19] Kim D. G. , Yim S. H. , Min E. K. , Choi M. C. , Lee J. G. , Kim M. S. , and Joo D. J. , Predicted Impact of the Model for End-Stage Liver Disease 3.0 in a Region Suffering Severe Organ Shortage, Journal of Korean Medical Science. (2023) 38, no. 35, e274, 10.3346/jkms.2023.38.e274, 37667579. 37667579 PMC10477074

[20] Barbosa B. C. , Santos L. A. R. , Daher G. H. R. M. , Martins D. L. , Perales S. R. , Gallani S. K. , Costa L. B. E. D. , Lago E. A. D. , Boin I. F. S. F. , Caserta N. M. G. , and Ataíde E. C. , Clinical Impact of the Model for End Liver Disease (MELD) Score on the Presence of Microvascular Invasion and on the Postoperative Outcome in Patients Undergoing Liver Transplantation, Revista do Colégio Brasileiro de Cirurgiões. (2021) 48, no. 48, e20212997, 10.1590/0100-6991e-20212997, 34932735. 34932735 PMC10683444

[21] Lee H. , Yoon S. , Oh S. Y. , Shin J. , Kim J. , Jung C. W. , and Ryu H. G. , Comparison of APACHE IV With APACHE II, SAPS 3, MELD, MELD-Na, and CTP Scores in Predicting Mortality After Liver Transplantation, Scientific Reports. (2017) 7, no. 1, 10884, 10.1038/s41598-017-07797-2, 2-s2.0-85029101736, 28883401. 28883401 PMC5589917

[22] Angus D. C. , Clermont G. , Kramer D. J. , Linde-Zwirble W. T. , and Pinsky M. R. , Short-Term and Long-Term Outcome Prediction With the Acute Physiology and Chronic Health Evaluation II System After Orthotopic Liver Transplantation, Critical Care Medicine. (2000) 28, no. 1, 150–156, 10.1097/00003246-200001000-00025, 2-s2.0-0033972117, 10667515. 10667515

[23] Pan H. C. , Jenq C. C. , Lee W. C. , Tsai M. H. , Fan P. C. , Chang C. H. , Chang M. Y. , Tian Y. C. , Hung C. C. , Fang J. T. , Yang C. W. , and Chen Y. C. , Scoring Systems for Predicting Mortality After Liver Transplantation, PLoS One. (2014) 9, no. 9, e107138, 10.1371/journal.pone.0107138, 2-s2.0-84933073884, 25216239. 25216239 PMC4162558

[24] Chung I. S. , Park M. , Ko J. S. , Gwak M. S. , Kim G. S. , and Lee S. K. , Which Score System Can Best Predict Recipient Outcomes After Living Donor Liver Transplantation?, Transplantation Proceedings. (2012) 44, no. 2, 393–395, 10.1016/j.transproceed.2012.01.064, 2-s2.0-84863233351, 22410025. 22410025

[25] Basile-Filho A. , Nicolini E. A. , Auxiliadora-Martins M. , and Silva O. D.Jr., The Use of Perioperative Serial Blood Lactate Levels, the APACHE II and the Postoperative MELD as Predictors of Early Mortality After Liver Transplantation, Acta Cirúrgica Brasileira. (2011) 26, no. 6, 535–540, 10.1590/s0102-86502011000600021, 2-s2.0-82355168959, 22042120. 22042120

